# 

**Published:** 1889-12-14

**Authors:** 


SATURDAY, DECEMBER 14th, 1889.

**Registration of Midwives and Nurses** - - - 161
**Words of Consolation**—Thick Darkness - - - - 162
**The Registration of Midwives and Nurses.**—Important Decision of the General Council of Medical Education and Registration - - - - - - - 163
**The Doctor's Pulpit.**—Physiological Candles - - 165
**The Oppression of the Medical Practitioner.** By W. MOORE, M.R.C.S. - - - - - - - - 166
**A Word for Daudet** - - - - - - - - 166
**Everybody's Page** - - - - - - - - 167
**Notes and News** - - - - - - - - - 168
**Annotations.**—Epidemic Influenza—The Parish Doctor—The Judge and the Professor - - - - - - - 170
**The Practitioner's Outlook and Retrospect:**
MEDICINE—Enteric Fever in India—Hypnotism - - - 171
SURGERY—Injuries from Electricity—Minor Operations—Surgical Cases - - - - - - - - - 172
THERAPEUTICS—Meat Diet—Treatment of Diphtheria - 173
THE PRACTITIONER'S MIRROR OF CASES—Scarlet Fever in a Family of Five Children - - - - - - 173
NEW DRUGS, APPLIANCES, AND THINGS MEDICAL—Æsculap Mineral Water—Antiseptic Dressings - - - - 174
**The Emigrant's Hospital at Ward Island** - - 175
**Editor's Letter-Box**—Professional Pressure - - 175
**Fallow Crops.**—The Recent Hospital Dispute - - 176
**Scraps and Gleanings** - - - - - - - - 176

**EXTRA SUPPLEMENT—THE NURSING MIRROR.**

**En Passant.**—Church Army Nurses—The Gratitude of a Dane—A Nurses' Conversazione—Army Nurses—Northampton Institute—Epsom District Nurse—Pauper Nurses No More—Death on Duty - - - - - - xlv
**Among the Nurse-Dolls at Charing Cross** - - xlvi
**Examination Questions** - - - - - - - xlvi
**The National Pension Fund for Nurses** - - xlvii
**Everybody's Opinion.**—Overworked Nurses—"An A B C. My Ideal Nurse" - - - - - - - - xlvii
**Notes and Queries** - - - - - - - - xlviii
**Presentations—Vacancies** - - - - - - - xlviii
**Amusements and Relaxation** - - - - - - xlviii

